# Three Dimensional Organization of the Nucleus: adding DNA sequences to the big picture

**DOI:** 10.1186/s13059-015-0751-9

**Published:** 2015-08-29

**Authors:** David M. Gilbert, Peter Fraser

**Affiliations:** Department of Biological Science, Florida State University, Tallahassee, FL 32306-4295 USA; Center for Genomics and Personalized Medicine, Florida State University, Tallahassee, FL 32306-4295 USA; Nuclear Dynamics Programme, The Babraham Institute, Cambridge, CB22 3AT UK

Fifteen years after the complete sequencing of the human genome, our understanding of how that sequence information is packaged within the cell nucleus and the significance of that packaging to the proper spatial and temporal regulation of gene expression is still poorly understood. While the breakthroughs in understanding the first level of packaging, the nucleosome, occurred at the end of the twentieth century, higher levels of interphase chromatin packaging in cell nuclei have remained unverifiable. Researchers had to rely on a combination of microscopic methods to either look anonymously at chromatin by electron or light microscopy or examine the spatial arrangement of a few specific loci by fluorescence in situ hybridization [1]. Mapping three dimensional structure onto the genome map remained a formidable gap. Recent advances in mapping the contact points between segments of chromatin in intact cells using variants of the chromatin conformation capture (3C) method have provided robust means to study large-scale chromatin folding, from multi-kilobase-scale loops that connect promoters and enhancers [2, 3], through the organization of megabase-scale chromosomal domains [4–6], to complete chromosome tertiary structures and inter-chromosomal interactions [7]. This has permitted a fresh appreciation of how specific expression patterns correlate with, and in some cases depend upon, three dimensional structures [8–10]. This has been complemented by advances in microscope technology that have dramatically increased the resolution available in 3D FISH approaches [11].

This special issue of *Genome Biology* on the three dimensional organization of the nucleus highlights several new developments in 3D genome structure. A good overview of the field is provided in a high level review by Britta Bouwman and Wouter de Laat of the discoveries that have been made at various scales of analysis of chromatin interactions, from loops to topologically-associating domains, and sub-nuclear compartments [12]. A review by Chang Liu and Detlef Weigel discusses higher order chromosome structure in plants and the similarities to, and some fundamental differences from, the situation in mammalian cells [13]. Ferhat Ay and William Noble outline the computational methods that have been developed to analyze the genome-wide derivative of 3C, Hi-C [14]. Also on the theme of methods, in a research paper in this issue, Nagano *et al*., systematically compare Hi-C results using different ligation modes [15].

Essential to the three dimensional organization of chromatin are the machineries that process the genetic information: replication, recombination, repair, transcription and RNA processing machineries. Understanding how the 3D organization of chromatin influences, and is influenced by, these machineries is likely central to our understanding of how cells access and interpret genetic information. In particular, with the wealth of new transcriptome and epigenomic data, an important goal has been to determine which putative functional elements interact with which outputs. For example, linking promoters to their regulatory enhancers is central to understanding transcriptional regulation and to link GWAS SNPs found in regulatory elements to the affected genes [16, 17]. In this regard, Fortin and Hansen [18] and Huang *et al*. [19] introduce novel methods to predict chromosome organization from a set of epigenetic chromatin marks, while Sahlén *et al*. introduce a method to identify enhancer-promoter interactions by capturing the sequences from Hi-C libraries that are specific to promoters (HiCap) [20], focusing the dataset on sequences that interact with promoters.

In female mammals, one of the two X chromosomes is almost completely inactivated to balance the double dose of the genes on this chromosome as compared to males. As part of this silencing process, the inactive X takes on a very unusual compact structure that is explored in a series of papers. Philip Avner and colleagues provide a review of what is known about *Xist*, the lncRNA mediating inactivation [21]. Deng *et al*. explore the bipartite structure of the inactivated X chromosome [22], and Marks *et al*. investigate the time course of inactivation of specific genes during differentiation [23]. Together these studies link structure to function during dosage compensation.

The issue also contains a series of articles highlighting other aspects of the three dimensional ‘nucleome’. A pair of research articles illustrates how these three dimensional concepts are put into a functional context to regulate transcription. Rafique *et al*. report on estrogen-induced changes in chromatin architecture [24], and Pugacheva *et al*. show how BORIS cooperates with its paralog CTCF to shape gene expression and chromatin architecture in cancer cells and germ cells [25]. Understanding how chromatin is organized in three dimensions in the nucleus has opened new avenues for understanding how double stranded DNA breaks are repaired, and Burman *et al*. introduce new high-throughput microscopy approach to detect chromosomal translocations resulting from aberrant repair of DNA breaks [26]. Finally, Susan Gasser and colleagues review what is among the most prominent and well-studied geographical landmarks in the nucleus, the nuclear lamina, and how chromosomes associate with this structure [27].

It has long been known that the simple linear model of genes on a chromosome, activated by upstream promoters, is not the complete picture of gene control. The recent explosion of 3C and Hi-C data, and 3D FISH, is beginning to show us the complexity of how genes, regulatory elements and chromosomes interact. As ever, with a new technology, the excitement of the new results is tempered by the realization of its limitations and how little we still know. *Genome Biology* is excited to publish this special issue now, and looks forward to publishing related articles in the future that build on the platform provided by these and other studies to refine our knowledge of the organization of the nucleus and its impact on genome function.
